# MITF has a central role in regulating starvation-induced autophagy in melanoma

**DOI:** 10.1038/s41598-018-37522-6

**Published:** 2019-01-31

**Authors:** Katrin Möller, Sara Sigurbjornsdottir, Asgeir O. Arnthorsson, Vivian Pogenberg, Ramile Dilshat, Valerie Fock, Solveig H. Brynjolfsdottir, Christian Bindesboll, Margret Bessadottir, Helga M. Ogmundsdottir, Anne Simonsen, Lionel Larue, Matthias Wilmanns, Vesteinn Thorsson, Eirikur Steingrimsson, Margret H. Ogmundsdottir

**Affiliations:** 1 Department of Biochemistry and Molecular Biology, Biomedical Center, Faculty of Medicine, University of Iceland, Sturlugata 8, 101 Reykjavik, Iceland; 2 European Molecular Biology Laboratories, Notkestrasse 85, 22761 Hamburg, Germany; 3 Institute of Basic Medical Sciences and Centre for Cancer Cell Reprogramming, Institute of Clinical Medicine, Faculty of Medicine, University of Oslo, 1112 Blindern, 0317 Oslo, Norway; 4 Institut Curie, PSL Research University, INSERM U1021, Normal and Pathological Development of Melanocytes, Orsay, France; 5 Université Paris-Sud, Université Paris-Saclay, CNRS UMR 3347, Orsay, France; 6 Equipe Labellisée Ligue Contre le Cancer, Orsay, France; 7 Institute for Systems Biology, 401 Terry Avenue North, Seattle, WA 98109 USA

## Abstract

The MITF transcription factor is a master regulator of melanocyte development and a critical factor in melanomagenesis. The related transcription factors TFEB and TFE3 regulate lysosomal activity and autophagy processes known to be important in melanoma. Here we show that MITF binds the CLEAR-box element in the promoters of lysosomal and autophagosomal genes in melanocytes and melanoma cells. The crystal structure of MITF bound to the CLEAR-box reveals how the palindromic nature of this motif induces symmetric MITF homodimer binding. In metastatic melanoma tumors and cell lines, MITF positively correlates with the expression of lysosomal and autophagosomal genes, which, interestingly, are different from the lysosomal and autophagosomal genes correlated with TFEB and TFE3. Depletion of MITF in melanoma cells and melanocytes attenuates the response to starvation-induced autophagy, whereas the overexpression of MITF in melanoma cells increases the number of autophagosomes but is not sufficient to induce autophagic flux. Our results suggest that MITF and the related factors TFEB and TFE3 have separate roles in regulating a starvation-induced autophagy response in melanoma. Understanding the normal and pathophysiological roles of MITF and related transcription factors may provide important clinical insights into melanoma therapy.

## Introduction

Autophagy is a major intracellular degradation pathway that occurs at basal levels in all cells and is necessary for maintaining cellular homeostasis by degrading protein aggregates, long-lived proteins, lipids and malfunctioning organelles. Macroautophagy (hereafter referred to as autophagy) involves the formation of a double membrane structure (the phagophore) that engulfs cytoplasmic material and closes to form an autophagosome, which fuses with the lysosome, leading to degradation of the sequestered material. Autophagy can be induced by various stress conditions, such as nutrient deprivation, hypoxia or infection. The autophagy process generates amino acids for protein synthesis and lipids for β-oxidation, thereby producing new building material and energy in the form of ATP for cell survival^1^. Autophagy plays a major role in both tumor prevention and tumor formation, and has been shown to promote metastasis by enhancing tumor cell fitness in response to environmental stresses during the metastatic process^2,3^.

The MiT/TFE transcription factor family, consisting of Microphthalmia-associated transcription factor (MITF), TFEB, TFE3 and TFEC, belongs to the MYC superfamily of basic helix-loop-helix leucine zipper (bHLH-ZIP) proteins. The basic domains are involved in binding DNA whereas the HLH and Zip domains are important for the dimerization. The DNA binding and dimerization domains of the MiT/TFE proteins are highly conserved^4^ and the members bind DNA as homo- and heterodimers with each other, but not with other bHLH-ZIP proteins such as MYC, MAX or USF^5^. The MiT/TFE factors specifically bind to E- (CANNTG) and M-box (TCATGTGA) elements in the promoter regions of their target genes^6^. They are found in most vertebrate species^7^ and share a common ancestor in *D. melanogaster* (*Mitf*)^8^ and *C. elegans* (*HLH-30*)^7^. The central role of the sole Mitf factor in the fruit fly is to regulate the expression of all genes encoding subunits of a functional vacuolar H+-ATPase (v-ATPase)^9–11^, an ATP-dependent proton pump that is responsible for the acidification of the lysosome. Proper pH regulation of the lysosome is important for cellular processes such as degradation in lysosomes through autophagy^12^. Genes involved in lysosomal biogenesis and autophagy have been shown to be transcriptionally regulated by TFEB and TFE3 via their binding to the E-box type CLEAR (TCACGTGA) element^13–17^. Mammalian MITF on the other hand, is mainly known to regulate melanosome formation and melanin production in melanocytes, a process also based on proper lysosomal pH regulation^18^. Additionally, MITF regulates genes involved in cell proliferation and survival and it plays an important role in melanoma, where it has been suggested to act as a lineage-specific oncogene^19^.

Recently, MITF has been suggested to play a role in the regulation of lysosomal biogenesis and autophagy via transcriptional regulation of lysosomal and autophagosomal genes^20,21^. Interestingly, these genes are expressed at higher levels in melanoma than in most other cancers and melanomas have been shown to be highly dependent on lysosomal and autophagic activity for both prevalence and progression^22–24^. Here we show that MITF binds, with high affinity, to promoters of a subset of lysosomal and autophagosomal genes in melanoma cells. MITF knockdown reduces the autophagy response to starvation in both melanocytes and melanoma cells, whereas MITF overexpression leads to an increased number of autophagosomes. In metastatic melanoma tumours, *MITF* mRNA levels correlate with a subset of lysosomal and autophagosomal genes, that is different to the subset of genes regulated by TFEB and TFE3. These results suggest a distinct role for MITF in regulating stress-induced autophagy in melanoma cells.

## Results

### MITF binds the promoters of lysosomal and autophagosomal genes

**Figure 1:**
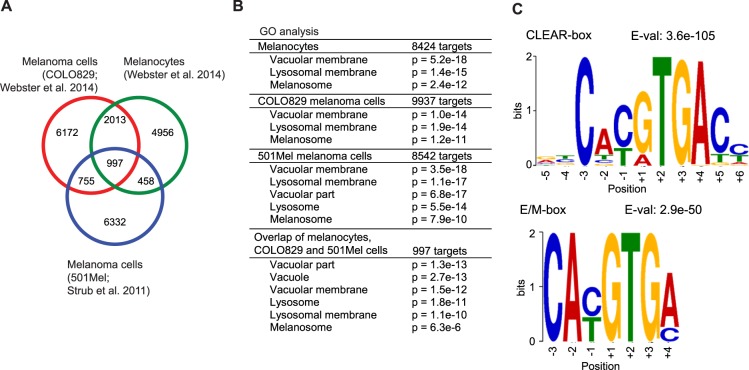
MITF binds lysosomal and autophagosomal genes in melanoma cell lines. (**A**) Analysis of MITF ChIP sequencing data from primary melanocytes^27^ and the two melanoma cell lines COLO829^27^ and 501Mel^25^. The Venn diagram shows the number of MITF binding sites in each dataset. (**B**) Gene ontology analysis for each MITF ChIP sequencing dataset. (**C**) Motif analysis of the shared 997 binding sites.

Experimental evidence has shown that MITF regulates expression of genes involved in diverse cellular processes in the melanocyte lineage, including pigment production^25,26^. To characterize which genes are mainly bound by MITF in melanocytes and melanoma cells, we analysed previously published MITF ChIP sequencing data from primary human melanocytes (NHEM) and from two human melanoma cell lines; COLO829 and 501mel^25,27^. Binding sites were assigned to genes using the GREAT software^28^. Comparison of MITF binding sites in these three data sets revealed 997 overlapping sites, corresponding to 940 common genes in all three cell types (Fig. 1A). Gene ontology (GO) analysis of the MITF bound genes revealed an enrichment of lysosomal genes, in addition to melanosomal genes (Fig. 1B). GO analysis showed a significant presence of lysosomal and melanosomal genes among the overlapping genes (Fig. 1B), suggesting that these are common targets of MITF in the melanocyte lineage. Motif analysis of these 997 overlapping MITF binding sites in the different cell lines revealed the presence of a CLEAR-box element in addition to E- and M-box elements (Fig. 1C). To verify that MITF can bind to specific melanosomal and lysosomal genes in a human melanoma cell line, we performed ChIP on endogenous MITF in 501Mel cells, followed by qRT-PCR. Indeed, MITF binds to the promoters of *MLANA* (melanosomal gene) as well as to several lysosomal and autophagosomal genes, such as *LAMP1* and *MAP1LC3B (LC3B)*, in these cells (Sup. Fig. 1A).

### MITF binds to the CLEAR-box element in a symmetric manner

**Figure 2:**
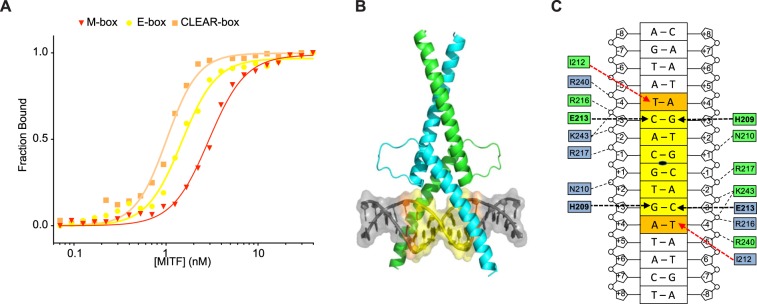
MITF binds the CLEAR-box motif in a symmetric manner. (**A)** Representative titration curves of each fluorescein-labeled oligonucleotide M-box (red), E-box (yellow) and CLEAR-box (orange), with MITF. The calculated dissociation constants are given in Table 1. (**B**) Crystal structure of MITF in the presence of 16-bp oligonucleotides covering the CLEAR-box motif. Proteins are shown in cartoon representation and DNA is illustrated in hybrid cartoon/surface representation. Color codes: central E-box motif in yellow; bases in positions -4 that are different in CLEAR- and E-box are highlighted in orange. (**C**) Schematic presentation of the MITF/DNA interactions with the CLEAR-box motif. Base-specific interactions are highlighted in bold and remaining hydrogen bond interactions in regular characters. Hydrogen bonds are indicated with black dashed arrows. I212 van der Waals contacts with thymidine -4 are depicted in red.

We have previously shown that different amino acid-DNA contacts are involved when MITF binds to the M-box element than when it binds to the palindromic E-box element^29^. Using fluorescence anisotropy, we compared the binding affinity of MITF to the M-, E- and CLEAR-box elements, revealing similar affinity to all motifs, with a slight preference for the CLEAR-box (Fig. 2A, Table 1). We further measured the MITF-mediated activation of M-, E- and CLEAR-box elements in HEK293T cells using a luciferase trans-activation assay. Our results show that MITF activates expression from all three elements, with a slight preference for the E- and CLEAR-box over the M-box (Sup. Fig. 1B).

**Table 1:** Quantitative determination of MITF binding affinity for various DNA elements by fluorescence anisotropy, measured as K_D_ ± standard deviations (nM).

DNA	M-box	E-box	CLEAR-box
K_D_	2.6 ± 0.4	1.5 ± 0.3	0.9 ± 0.3

We solved the crystal structure of the complex formed by MITF and the CLEAR-box DNA motif (Fig. 2B). In contrast to what we previously observed in the MITF structures in presence of M- and E-box elements^29^, the MITF/CLEAR assembly is symmetric and associated with a crystallographic two-fold axis (Fig. 2B). In addition to base-specific and phosphate backbone hydrogen bonds, which are similar to the ones observed in the previous study^29^, the binding is reinforced by the van der Waals contact between I212 and hydrophobic patches at the surface of the DNA including the methyl moiety of the thymidine in position -4 of both half-sites of the CLEAR motif (Fig. 2C, Sup. Table 1). In contrast, this base is present only in one half-site of the asymmetric M-box, and this could explain that the affinity of MITF for the M-box is slightly lower than for the CLEAR-box. Taken together, these data show that MITF is fully able to recognize the CLEAR-box element and therefore activate the expression of lysosomal and autophagy genes through the binding of their promoters.

### MITF and TFE3 negatively correlate in metastatic melanoma tumors

**Figure 3:**
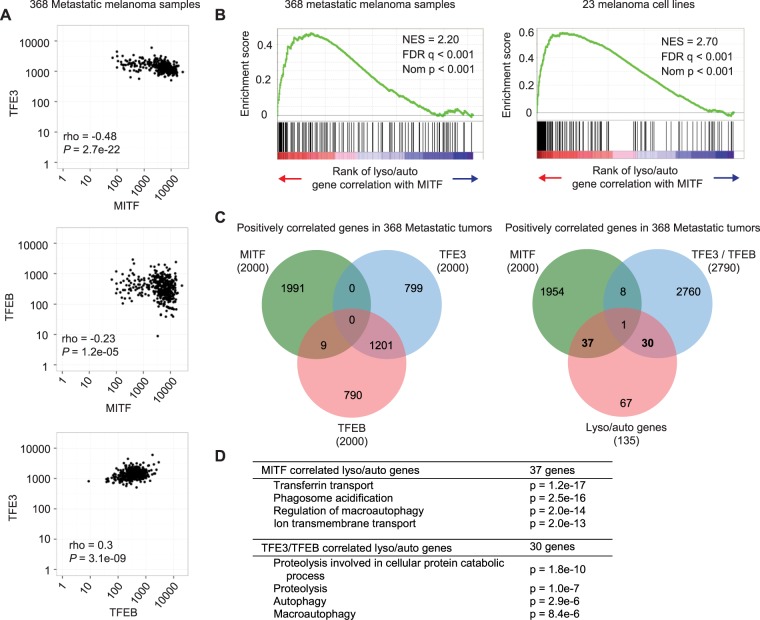
Analysis of the correlation of *MITF, TFEB* and *TFE3* in melanoma tumors. (**A**) Cross comparison of expression levels of *MITF*, *TFEB* and *TFE3* in 368 TCGA metastatic melanoma tumor samples. (**B**) Gene set enrichment analysis of expression data from 368 metastatic melanoma samples from TCGA and 23 melanoma cell lines^33^. Genes were ranked based on correlation with MITF expression and lysosomal and autophagosomal genes highlighted. Normalised Enrichment Score (NES), False Discovery Rate (FDR) and Nominal p values are shown. (**C**) Left: Venn diagram representing top 2,000 positively correlated genes with MITF, TFEB or TFE3 expression in 368 metastatic melanoma samples. Right: Venn diagram representing lysosomal and autophagosomal genes (135 genes), 2,000 top positively correlated genes with MITF, as well as the top 2,000 positively correlated genes with TFE3 and/or TFEB (2,790 unique genes; 1,201 genes are positively correlated with both TFE3 and TFEB). (**D**) GO analysis on lysosomal and autophagosomal genes that uniquely correlated with MITF and on lysosomal and autophagosomal genes that uniquely correlated with TFEB or TFE3.

As autophagy levels have been shown to be high in melanoma, we investigated the involvement of MITF in regulating expression of lysosomal and autophagosomal genes in these tumors. Using RNA sequencing data from 368 metastatic melanoma tumors from The Cancer Genome Atlas (TCGA)^30^ we analysed the correlation between *MITF* and these genes. In terms of overall expression level, *MITF* had a 4- and 15-fold higher mRNA expression than that of TFEB and TFE3, respectively, in the metastatic tumors (Sup. Table 2). We included TFEB and TFE3 in our subsequent analyses but excluded the fourth related factor, TFEC, due to its very low expression levels in these tumors (Sup. Table 2). Interestingly, *TFEB* and *TFE3* negatively correlate with *MITF* expression in the metastatic melanoma tumors (Fig. 3A). All genes were ranked according to the correlation of their expression levels with that of *MITF*. The genes bound by MITF in all three MITF ChIP sequencing data sets (Fig. 1A) have higher correlation with *MITF* in these tumors than do other genes (Sup. Fig. 2A). This indicates that the expression of genes bound by MITF in melanocytic and melanoma cell lines are positively regulated by MITF in melanoma tumors. Gene Set Enrichment Analysis (GSEA)^31,32^ performed on TCGA expression data from metastatic melanoma tumors revealed that the ranks of correlation of *MITF* with lysosomal and autophagosomal genes were higher than ranks expected by chance (Fig. 3B, Sup. Fig. 2B). The same was observed for the correlation of both *TFEB* and *TFE3* with lysosomal and autophagosomal genes (Sup. Table 3, Sup. Fig. 2B). Whereas all three factors positively associate with lysosomal and autophagosomal genes, only *MITF* levels positively correlate with those of melanosomal genes (Sup. Table 3, Sup. Fig. 2B). In fact, *TFE3* negatively correlates with the expression of melanosomal genes in the metastatic tumors, indicating a specific role for MITF in regulating melanosomal genes.

Next we analysed expression data from 23 melanoma cell lines^33^. GSEA revealed a strong positive correlation of *MITF* with both melanosomal and lysosomal genes in these cell lines (Fig. 3B, Sup. Table 3, Sup. Fig. 3A,B), whereas *TFEB* showed no correlation with these genes, and *TFE3* negatively correlated with the melanosomal and lysosomal genes. It is important to keep in mind that *MITF* expression is about 50-fold higher than that of *TFEB* or *TFE3* in these cells (Sup. Table 2).

In metastatic melanoma tumors, *MITF*, *TFEB* and *TFE3* all show elevated correlation with lysosomal and autophagosomal genes (Fig. 3B, Sup. Fig. 2B). However, the expression of *MITF* negatively correlates with *TFEB* and *TFE3* expression (Fig. 3A), raising the question whether MITF regulates a different set of lysosomal genes than the other factors in melanoma. Comparing the correlation of the expression of each of the transcription factors MITF, TFEB or TFE3, with the expression of genes involved in lysosomes and autophagosomes, shows that *MITF*-positively correlated genes have a negative correlation with *TFE3* or *TFEB* and vice versa (Sup. Fig. 3C). Analysing the 2,000 genes which separately exhibited the highest positive correlation with *MITF*, *TFE3* or *TFEB* in the metastatic tumors, showed that whereas *TFEB* and *TFE3* share an overlap of 1,201 correlated genes, *MITF* correlated genes do not overlap with any *TFE3* correlated genes and only with 9 *TFEB* correlated genes (Fig. 3C). GO analysis on the 2,000 genes with the highest positive correlation with each factor revealed that *MITF* positively correlates with genes involved in mitochondrial function, membrane transport and endosomal acidification (Sup. Table 4). Interestingly, *TFEB* and *TFE3* show strong correlation with genes involved in the immune response in these tumors, implying a distinct role for these factors in melanoma tumors.

Among the top 2,000 genes that positively correlated in expression with *MITF*, *TFEB* and *TFE3*, only one lysosomal and autophagosomal gene (*GM2A*) was correlated with both MITF and TFEB, whereas 37 such genes were uniquely correlated with *MITF* and 30 genes uniquely correlated with *TFEB* and/or *TFE3* (Fig. 3C,D). We performed a GO analysis on these uniquely correlated lysosomal and autophagy genes and interestingly, MITF mainly correlated with genes involved in phagosome acidification and membrane transport (Fig. 3D). *TFEB* and *TFE3* on the other hand correlated with genes involved in proteolysis and autophagosomal formation. MITF thus has a regulatory role within lysosomal function and autophagy in melanoma tumors, distinct from the related TFEB and TFE3 factors.

### MITF regulates starvation-induced autophagy in melanoma cells

**Figure 4:**
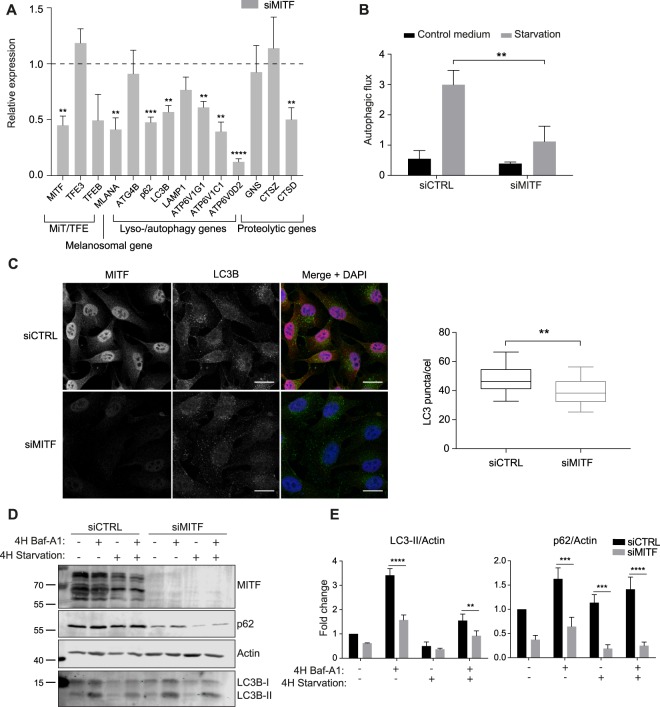
MITF regulates starvation-induced autophagy in SkMel28 melanoma cells. (**A**) Relative quantitative reverse transcription PCR (qRT-PCR) analysis of selected targets in SkMel28 cells treated with siMITF compared with siCTRL (siCTRL is normalised to 1 for all targets and not shown). An average of 3 independent experiments is shown. Error bars represent SEM, *P < 0.05, **P < 0.01, ***P < 0.001, ****P < 0.0001. (**B**) Degradation of long-lived proteins measured by ^14^C labelled Valine in SkMel28 cells treated with siMITF compared with control siRNA, incubated with or without Baf-A1 while grown in either normal culture medium or starved in HBSS for 4 hours. Each bar represents the difference of degradation in untreated and Baf-A1 treated cells, thus representing degradation by autophagy. An average of three independent experiments is shown. Error bars represent SEM, **P < 0.01. (**C**) Immunostaining of SkMel28 cells treated with siMITF or siCTRL, showing MITF (red), LC3B (green), and DAPI (blue) staining. Scale bar is 20 µm. Boxplot represents number of LC3B puncta per cell. **P < 0.01. (**D**) Immunoblot of protein lysate from siMITF and siCTRL treated SkMel28 cells cultured in normal medium or starved in HBSS for 4 hours with or without 100 nM Baf-A1. The blots were stained with C5 anti-MITF, anti-Actin, anti-p62 and anti-LC3B antibodies. The figure is representative of three independent experiments. Full scan of blots are found in Supplementary Fig. 4. (**E**) Quantification of the intensity of LC3B-II and p62 bands from immunoblot in C, normalised to Actin band intensity. An average of three independent experiments is shown. Error bars represent SEM, **P < 0.01.

To determine if MITF is involved in autophagy regulation in melanoma cells, we performed short-term knockdown of MITF in the human melanoma cell line SkMel28. Treating cells with siRNA against *MITF* (siMITF) resulted in about 50% reduced *MITF* mRNA expression and almost a complete removal of MITF protein levels, compared to cells treated with control siRNA (siCTRL; Fig. 4A,D). The melanosomal gene *MLANA* as well as the autophagy genes p62 and *LC3B*, genes encoding for v-ATPase subunits *ATP6V1G1*, *ATP6V1C1* and *ATP6V0D2*, and the lysosomal degradation enzyme gene *CTSD* were all decreased in expression in the MITF depleted cells (Fig. 4A). To monitor if *MITF* knock-down affects autophagic flux, the degradation of long-lived proteins, labelled with ^14^C-Valine, was measured in nutrient-rich and starvation conditions in the absence or presence of Bafilomycin-A1 (Baf-A1), an inhibitor of autophagic degradation. We observed no difference in total autophagic degradation of long-lived proteins when cells were cultured in normal medium, whereas autophagic degradation was decreased upon starvation in siMITF treated SkMel28 cells compared to siCTRL treated cells (Fig. 4B).

The conjugation of LC3B to phosphatidylethanolamine in the autophagosomal membrane is commonly used as an autophagy marker, and can be analysed by fluorescent imaging as lipidated LC3 forms distinct puncta in the cytoplasm. Comparing LC3B antibody staining of siMITF and siCTRL treated cells revealed fewer LC3B positive puncta in the MITF depleted cells (Fig. 4C). Lipid-bound LC3 can also be analysed by Western blotting, as the lipid-bound LC3B (LC3B-II) migrates faster on an SDS gel than the non-lipidated cytosolic LC3B (LC3B-I). After blocking autophagic degradation with Baf-A1, both in normal and starvation medium, we observed a reduction in LC3B-II levels in MITF depleted cells (Fig. 4D,E), indicating fewer autophagosomes. SQSTM1 (p62) levels were significantly lower after MITF depletion, and even though this would normally suggest increased autophagic degradation, p62 is a direct MITF target^25^ (Fig. 4A), complicating its use as a readout of autophagic activity. Nevertheless, p62 protein levels were increased after Baf-A1 treatment, but to a much lower extent than in control cells. Taken together, our results indicate that in SkMel28 melanoma cells, MITF knockdown decreases autophagosome formation, followed by a reduction in global autophagic degradation.

To determine if this effect on autophagosomal formation is specific to melanoma cells, we investigated whether knocking down MITF also affects autophagy in human primary normal epidermal melanocytes (NHEM). Analysis of melanosomal and lysosomal gene expression by qRT-PCR showed reduced expression of *MLANA* and the lysosomal and autophagosomal genes *ATG4B, p62, ATP6V1G1* and *ATP6V1C1*, as well as proteolytic genes *GNS* and *CTSD*, upon *MITF* knockdown (Sup. Fig. 5A). No difference was detected in the degradation of long-lived proteins when the cells were cultured in normal medium. However, autophagic flux was decreased upon starvation of the NHEM cells treated with siMITF compared with a control siRNA (Sup. Fig. 5B). Using Western blot analysis of LC3B lipidation, we observed a decrease in LC3B-II upon *MITF* knockdown when grown in normal medium (Sup. Fig. 5C,D), indicating that fewer autophagosomes are being formed. Consistent with the results in SkMel28 cells, p62 levels were significantly lower in the MITF depleted NHEM cells, both in normal and starvation medium. Taken together, these data suggest that MITF knockdown results in decreased global starvation-induced autophagic degradation in both NHEM melanocytes and SkMel28 melanoma cells.

**Figure 5:**
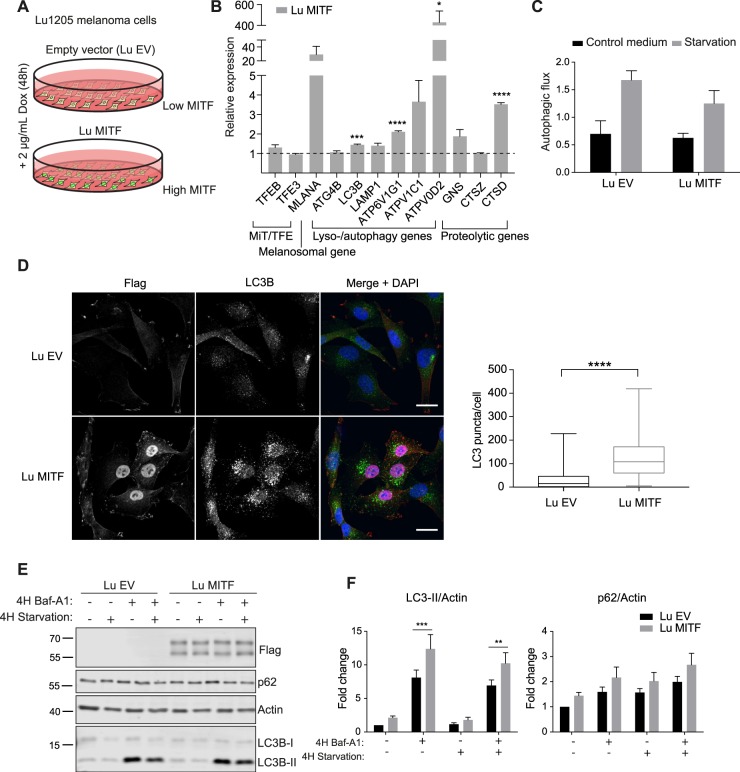
MITF overexpression increases the number of autophagosomes in Lu1205 melanoma cells. (**A**) An inducible stable Lu1205 melanoma cell line was generated, carrying a construct which allows doxycycline induced expression of MITF-M-Flag using the reverse tetracycline-system (Lu MITF). A comparable cell line carrying an empty vector (Lu EV) was used as a control. (**B**) qRT-PCR analysis of selected targets in the Lu1205 overexpression cells after 48 hours of induction, compared to the EV control cell line (EV is normalised to 1 for all targets and not shown). An average of 3 independent experiments is shown. Error bars represent SEM, *P < 0.05, **P < 0.01, ***P < 0.001, ****P < 0.0001. (**C**) Degradation of long-lived proteins measured by ^14^C labelled Valine in MITF overexpressing Lu1205 cells (Lu MITF) and control cells (Lu EV), treated with or without Baf-A1 while grown in either normal culture medium or starved in HBSS for 4 hours. Each bar represents the difference of degradation in untreated and Baf-A1 treated cells, thus representing degradation by autophagy. An average of three independent experiments is shown. Error bars represent SEM. (**D**) Immunostaining of MITF overexpressing Lu1205 cells (Lu MITF) and control cells (Lu EV) showing Flag (red) and LC3B (green) staining. Scale bar is 20 µm. Boxplot represents number of LC3B puncta per cell. Only MITF positive cells were analysed in the MITF-induced cells. ****P < 0.0001. (**E**) Immunoblot of protein lysate from MITF overexpressing cells (Lu MITF) and control cells (Lu EV) cultured in normal medium or starved in HBSS for 4 hours with or without 100 nM Baf-A1. The blots were stained with anti-Flag, anti-Actin, anti-p62 and anti-LC3B antibodies. The figure is representative of six independent experiments. Full scan of blots are found in Sup. Fig. 4. (**F**) Quantification of the intensity of LC3B-II and p62 bands from immunoblot in D, normalised to Actin band intensity. An average of 6 independent experiments is shown. Error bars represent SEM, *P < 0.05, **P < 0.01.

To investigate whether increased MITF expression alters autophagy activity in melanoma cells, we used Lu1205 cells, a melanoma cell line with very low endogenous MITF expression^33^, to generate a stable cell line carrying a construct which allows doxycycline-induced activation of MITF-M using the reverse tetracycline-system (Fig. 5A). After 48 hours of doxycycline induction, expression levels of the lysosomal and autophagosomal genes *LC3B*, *ATP6V1G1*, *ATP6V0D2* and *CTSD* were increased (Fig. 5B). However, MITF overexpression did not affect autophagic flux, measured by the degradation of long-lived proteins (Fig. 5C). Nevertheless, comparing LC3B antibody staining of MITF overexpressing cells and control cells revealed an increase in the number of LC3B positive puncta in MITF expressing cells (Fig. 5D). Consistently, LC3B-II levels, measured by Western blotting, were higher after MITF overexpression, in both normal and starvation conditions (Fig. 5E,F), which suggests an increase in the number of autophagosomes. There was a small, non-significant increase in p62 levels upon MITF overexpression, presumably reflecting MITF mediated transcriptional regulation of p62 (Fig. 5E,F).

In summary, our results show that MITF knockdown decreases the expression of several lysosomal and autophagosomal genes and reduces the autophagic response to starvation in both melanoma cells and primary melanocytes. However, while overexpression of MITF in the MITF-low melanoma cells increases expression of several lysosomal and autophagosomal genes and results in increased autophagosome formation, MITF is not sufficient to drive a global increase in autophagy-mediated protein degradation.

## Discussion

MITF is highly expressed in melanocytes and is required for melanocyte development and function, including formation of melanosomes and melanin production. Here we show that MITF also plays a central role in regulating the autophagy response to starvation in melanoma cells. We show that MITF binds the CLEAR box element in the promoters of lysosomal and autophagy genes, previously characterized as a preferred motif for TFEB and TFE3^16^. The crystal structure of MITF bound to the CLEAR-box element revealed symmetric binding of the homodimer to DNA. In our previous study^29^, we showed that MITF is capable of binding efficiently to both M- and E-boxes, in contrast to other bHLHZip transcription factors such as MAX and MYC which recognize mainly the E-box type of motif^34^. Here we show that this ability relates to the van der Waals contacts between I212 and T-4 allowing optimal binding to the CLEAR-box. Indeed, MITF, TFE3 and TFEB have a perfectly conserved basic region^29^ and can all bind the CLEAR box.

Analysis of expression data from metastatic melanoma tumors suggests that MITF has a role that is distinct from the role of TFEB and TFE3 in these tumors. *MITF* expression correlates with the expression of melanosomal genes in metastatic melanoma samples as well as in a panel of human melanoma cell lines. This is in concordance with the previously described role of MITF. *MITF* expression also correlates with the expression of lysosomal and autophagosomal genes in metastatic melanoma samples, as do *TFEB* and *TFE3*. However, the expression of *TFE3* and *TFEB* negatively correlates with *MITF* expression in melanoma tumor samples. Interestingly, *MITF* correlates with genes required for endosomal trafficking and endosomal transporters, consistent with previous findings showing the involvement of MITF in the regulation of the v-ATPase^9^, whereas, *TFEB* and *TFE3* mainly correlate with degradation enzymes involved in proteolysis. Due to their common origin, these factors still share a group of target genes. However, after their divergence, the individual family members, MITF, TFEB and TFE3, each have acquired more specific roles by adding or losing target genes.

TFEB and TFE3 have been shown to regulate lysosomal and autophagosomal gene expression in response to several stress factors including starvation^35^. We have shown that MITF is involved in regulating this response in melanocytes and melanoma cells. We observed that MITF knockdown leads to reduced starvation-induced autophagy degradation in both melanocytes and melanoma cells, presumably because of less autophagosomal formation. Consistent with that, the expression of lysosomal and autophagosomal genes was decreased upon MITF knockdown. While overexpressing MITF increased the expression of lysosomal and autophagosomal genes and induced autophagosome formation, MITF induction did not result in increased autophagy degradation, indicating that alone, MITF is not sufficient to fully drive autophagic degradation. This is in agreement with a previous publication showing that MITF induction increases the formation of lysosomes that are not fully active^20^. It remains to be clarified whether all MiT/TFE factors need to be expressed for a full response.

When nutrients are abundant, TFEB, TFE3 and a long isoform of MITF (MITF-A) are detained in the cytoplasm through mechanistic target of rapamycin kinase (mTOR) mediated phosphorylation; mTOR complex1 (mTORC1) interacts with the N-terminus of these proteins in order to mediate phosphorylation^17,36–38^. During cellular stress or starvation, mTORC1 becomes inactive and the MITF/TFEB/TFE3 proteins are dephosphorylated resulting in their translocation to the nucleus^14,17,37^. In melanocytes and melanoma cells, the dominant isoform is MITF-M^39^ that lacks the N-terminal interaction site with mTORC1 and is therefore mostly nuclear under all conditions^38^. The role and importance of localisation and heterodimerization of MITF, TFEB and TFE3 in melanoma cells remains to be explored and may reveal why MITF regulates a distinct set of genes compared to the other factors.

In metastatic melanomas *TFEB* and *TFE3* positively correlate with the expression of genes required for the immune response. These factors have previously been suggested to have a role in the immune system^16,35,40,41^. This role of TFEB and TFE3 in immune response in melanoma needs to be explored further, as this raises important therapeutic questions about their role in immunotherapy, a key treatment option for melanoma.

It is becoming clear that misregulation of vesicular trafficking is one of the hallmarks of melanomas and that melanomas are dependent on lysosomal activity and autophagy^23,42^. Autophagy has even been linked to increased invasiveness in melanoma and is believed to be a key modulator of inflammation and immune responses in these cancers^18^. Our results further link the MiT/TFE factors to these processes and underline the need to understand better how to target this pathway in melanoma and other cancers.

## Materials and Methods

### Cell culture

Three human melanoma cell lines were used in this study, 501Mel cells (a gift from Ruth Halaban)^43^, SkMel28 cells (#HTB-72, ATCC) and Lu1205 (a gift from Meenhard Herlyn, Wistar Institute, Philadelphia). These cells were all grown in RPMI 1640 medium (#52400-025, GIBCO) supplemented with 10% fetal bovine serum (FBS #10270-106, GIBCO). The primary normal human epidermal melanocytes (NHEM) were purchased from PromoCell (#C-12402) and were grown in Melanocyte growth medium M2 (#C-24300, PromoCell). Cells were grown at 37 °C and 5% CO_2_ and medium was changed three times per week.

### Generation of inducible MITF overexpression cell lines

The Lu1205 cells were transfected with three piggybac vectors, containing a reverse-tetracyclin transcription activator (rtTA), a transposase and mouse *MITF-M-FLAG-HA* construct or an empty piggybac vector (EV). The piggybac vectors were a kind gift from Dr. Kazuhiro Murakami (Hokkaido University)^44^. Selection was performed with 0.5 mg/ml G418 (#10131-035, GIBCO) for 8 days to obtain stable cell lines and 1 μg/mL doxycycline was used for induction.

### RNAi treatment

Cells were cultured in a 6 well plate for 24 hours before transfection with appropriate siRNA. Cells were transfected with 25 μM siRNA and 1 μL transfection reagent DharmaFECT (#T-2001-02, Dharmacon) per mL of culture medium. Cells were cultured for 2 days before extracting RNA or protein. The siRNAs used for the procedure were an siRNA for human MITF (#4390824, ID s8792, Ambion) and a control siRNA (sequence: 5′-UUCUCCGAACGUGUCACGUdTdT-3′, Dharmacon) that has been previously used^45^.

### Protein extraction and immunoblotting

For protein extraction, cells were cultured in 6, 12 or 24 well plates and lysed with Laemmli buffer and boiled at 95 °C for 5 minutes. The samples were then run on 8% or 12.5% SDS gels and blotted onto a 0.2 μm PVDF membrane (#88520, Thermo Scientific). The membranes were blocked with 3% BSA in TBS-T (0.1% Tween 20 in TBS) for 1 hour at room temperature, and stained over-night at 4 °C with 5% BSA in TBS-T and appropriate primary antibodies. The following primary antibodies were used: MITF (MS771-PABX, Thermo Scientific), Flag-M2 (F3165, Sigma), LC3B (#2775, CST), p62 (#88588, CST) and Actin (MAB1501, Millipore and #4970, CST). Membranes were washed with TBS-T and stained for 1 hour at room temperature with appropriate secondary antibodies. The secondary antibodies used were: anti-mouse IgG(H + L) DyLight 800 conjugate (#5257, CST) and anti-rabbit IgG(H + L) DyLight 680 conjugate (#5366, CST). The images were captured using Odyssey CLx Imager (LI-COR Biosciences).

### Gene expression analysis using relative quantitative real-time PCR

Total RNA was extracted from cells using TRIzol reagent (#15596-026, Ambion), DNase treated using the RNase free DNase kit (#79254, Qiagen) and re-purified with the RNeasy Mini kit (#74204, Qiagen). The cDNA was generated with High-Capacity cDNA Reverse Transcription Kit (#4368814, Applied Biosystems). All procedures were performed according to manufacturer’s instructions. Primers were designed for each target gene using NCBI Primer BLAST (Sup. Table 5), and qRT-PCR was performed with SYBR-Green mix (#4438, Sigma-Aldrich) using an Applied Biosystems 7500 qPCR machine (annealing at 60 °C). The qRT-PCR reactions were performed using 5 ng cDNA per 20 µl reaction, in triplicates and relative gene expression was calculated with the D-ΔΔCt method^46^, using the geometric mean of β-Actin and human ribosomal protein lateral stalk subunit P0 (RPLP0) expression to normalise gene expression of target genes. Standard curves were made for each primer pair and the efficiency calculated using the formula E = 10[−1/slope].

### Immunostaining

Cells were seeded onto 8-well chamber slides (#354108 from Falcon) and cultured for 2 days. At day 2, cells were fixed for 20 minutes with 4% paraformaldehyde (PFA) in PBS. Cells were washed 3 times with PBS and blocked with blocking buffer (5% Normal goat serum and 0.3% Triton-X in PBS) for 1 hour at room temperature and stained over-night at 4 °C with the appropriate primary antibodies diluted in antibody buffer (1% BSA and 0.3% Triton-X in PBS). Primary antibodies were: MITF (MS771-PABX, Thermo Scientific), Flag-M2 (F3165, Sigma) and LC3B (#2775, CST). Cells were washed 3 times with PBS and stained for 1 hour at room temperature with the appropriate fluorescently labelled secondary antibodies (Thermo Scientific), diluted in antibody buffer. The wells were washed once with PBS, followed by DAPI staining (#D-1306, Life Technologies) and two additional washes with PBS only. Subsequently, slides were mounted in Fluoroshield (#F6182, Sigma-Aldrich) and images were acquired using an Olympus FC1200 confocal microscope. LC3B positive puncta were counted using the CellProfiler software and the number of puncta was normalised to the number of nuclei. For MITF-induced Lu1205 cells, LC3B positive puncta were only counted in MITF-FLAG positive cells.

### Luciferase transcriptional activation assay

HEK293T cells were seeded in a 96 well and transfected the following day with MITF-M (#38131, Addgene) and a pGL3-basic luciferase construct containing a modified Tyrosinase promoter with two M-(TCATGTGA), E-(CCACGTGC) or CLEAR-(TCACGTGA) box elements, or scrambled elements (ACCTTCAG/GTCTAGAT), along with an internal control construct, Renilla. A total of 110 ng of DNA were transfected per well, 33 ng per construct. 24 hours after transfection the cells were lysed using the Dual-Glo® Luciferase Assay System (#E2940, Promega) and the luminescence measured in a Modulus™II microplate multimode reader (Turner Biosystems). Data was analysed by normalising luciferase luminescence to Renilla luminescence (Luciferase/Renilla) and normalising luminescence of each transcription factor to empty vector by subtraction.

### Long-lived protein degradation assay

Cells were grown in 24 well plates for 48 hours in normal medium supplemented with 10 mM ^14^C-L-Valine. At day 2 cells were washed with warm PBS and grown in RPMI and 10% FBS supplemented with 10 mM L-Valine for 16 hours. At day 3 the medium was changed to (a) RPMI, 10 mM Valine and vehicle control (DMSO), (b) RPMI, 10 mM Valine and 100 nM Baf-A1, (c) HBSS (#14025-050, Gibco), 10 mM Valine and vehicle control (DMSO) or (d) HBSS, 10 mM Valine and 100 nM Baf-A1. After 4 hours, the supernatant was collected, 50% TCA added and proteins precipitated over-night at 4 °C. The cells were lysed with 0.2 M KOH over-night at 4 °C. The supernatant was centrifuged and moved to a new tube, the precipitate dissolved and moved to the same sample cell lysate. Both supernatant and lysate were moved to counting vials and mixed with 3 mL scintillation fluid (Ultima Gold #6013321, Perkin Elmer). ^14^C levels were measured in each sample using a Packard Liquid Scintillation Analyser. The % degradation was calculated by comparing the amount of ^14^C in the supernatant to the total ^14^C levels (supernatant and lysate). The autophagic flux was calculated by subtracting the % degradation of the Baf-A1 treated sample from the untreated sample, for each culture medium.

### Gene expression analysis from melanoma cell lines

The GSEA analysis was performed using the GSEA software from the Broad Institute^31,32^. The list of lysosomal and autophagosomal genes used has been described previously^21^ and the list of melanosomal genes was generated from melanosomal GO gene sets (GO:0042470, GO:0032438 and GO:0032400). These lists of 135 melanosomal genes and 132 lysosomal genes have an overlap of 11 genes.

### Chromatin Immunoprecipitation ChIP-qPCR

Chromatin immunoprecipitation was performed as described (Shaffer *et al*., 2008) with the following modifications: Thirty million cells were crosslinked with 0.4% formaldehyde, and chromatin was sheared by sonication using a probe sonicator (Epishear, Active Motif). Immunoprecipitation was performed with Protein G Dynabeads (10003D, Life technologies), with a total of 10 µg of anti-MITF antibody (Cosmo Bio Co., BAM-73-107-EX)^47^. Purified ChIP samples and corresponding input DNA were analysed by qRT-PCR using SYBR-Green mix (#4438, Sigma-Aldrich) on an ABI 7500 qPCR machine (annealing at 60 °C) using region specific primers (Sup. Table 6). The resulting qPCR data were then analysed as described above.

### ChIP-seq data analysis

MITF-ChIP-seq data with accession number GSE50681: SRX346923 (COLO829) and SRX346921 (Melanocyte) were downloaded from the SRA archive; 501Mel with accession number GSE61965. Raw reads were aligned to hg19 with bowtie1 with command -v 2 -m 1–best and peaks were called with MACS14. The Bioconductor package DiffBind was used to find overlapping peaks in different cell types, COLO829, 501Mel, and Melanocyte. Overlapping peak sets were annotated and GO analysis performed using GREAT^28^.

### Genome-wide gene expression data

23 melanoma cell lines were analysed with respect to gene expression (Agilent gene expression data as previously described)^33^. All available TCGA melanoma (SKCM) data were retrieved from the TCGA Data Coordinating Center on June 14, 2015 and processed through the TCGA pipeline at the TCGA Genome Data Analysis Center at the Institute for Systems Biology. After exclusion of Redacted cases, 109 primary tumor samples and 368 metastatic tumor samples remained, corresponding to 470 participants. We included the 368 metastatic tumor samples in our analysis. Gene expression matrices were generated for all samples with available (TCGA Level 3) gene expression values (from RNA-Seq, as RSEM values), resulting in a matrix for metastatic tumor samples (368 samples; 20,531 genes). Venn diagrams were plotted using jvenn software^48^. GO analysis was performed using the DAVID Bioinformatics Resources 6.8^49,50^.

### MITF purification and crystallization and X-ray structure determination

The following oligonucleotide was synthesized at METABION (Planegg/Steinkirchen, Germany) and annealed through incubation at 95 °C for 5 minutes, followed by a passive cooling step to room temperature: CLEAR-box: 5′- AGTATCACGTGATACT-3′.

The MITF/DNA complex was purified as previously reported^29^. Crystals grew from 0.9 M sodium malonate (pH 5.5). Crystals were soaked in cryo-solutions containing the crystallization mother liquor supplemented with 25% [v/v] glycerol, mounted onto a cryoloop (Hampton Research), and flash-cooled in liquid nitrogen. X-ray data were collected on the MASSIF-1 beamline at ESRF, Grenoble, France. Diffraction data were processed using XDS and scaled with AIMLESS from the CCP4 suite^51^. The structure was solved by molecular replacement using the program PHASER^52^ and refined with the PHENIX suite^53^ and REFMAC5^54^. The final model was built with COOT^55^. The stereochemical quality of the structures was assessed with MolProbity^56^. X-ray structure determination and refinement statistics are listed in Sup. Table S1. The atomic coordinates and structure factors have been deposited at the Protein Data Bank under the identification code 6G1L.

### Fluorescence anisotropy assay

The following fluorescein-labeled oligonucleotides were synthesized at METABION (Planegg/Steinkirchen, Germany) and annealed with complementary unlabeled oligonucleotides as described in the previous section:

CLEAR-box: 5′-GAGATCACGTGATGAC-3′-Fluorescein

E-box: 5′-GAGACCACGTGTTGAC-3′-Fluorescein

M-box: 5′-GAGATCATGTGTTGA C-3′-Fluorescein

Increasing concentrations of MITF proteins were incubated with the respective dsDNA oligonucleotides at a final concentration of 0.5 nM at 25 °C for 5 minutes in 10 mM Tris/HCl pH 7.5, 300 mM NaCl, 0.01% TRITON-X100, and 0.1 mg/mL BSA. Fluorescence anisotropy was then measured using an Infinite M1000 plate reader (TECAN) using the excitation diode at 470 nm and detecting the emitted light at 530 nm. Binding data were analysed using the GraphPad Prism software. Binding profiles were fitted using a simple model assuming a stoichiometry of one MITF dimer per double stranded DNA fragment. K_D_ values reported in Table 1 correspond to the means of three independent measurements and the +/− error numbers represent the standard deviations.

### Statistical analysis

Results from three or more independent experiments are presented as the mean with standard error of the mean (SEM). Graphpad Prism 7 was used for statistical analysis. Statistics for LC3B-positive puncta from confocal images were calculated using a two-tailed, unpaired t-test. LC3B positive puncta were counted using the CellProfiler software (80–350 cells analysed per cell line). Statistics of the long-lived protein degradation (LLPD) assay, immunoblotting and immunostaining experiments and qRT-PCR analysis for knockdown and overexpression experiments, were calculated using multiple t-tests and a false discovery rate of 5%. Significant differences of the mean are indicated as *P < 0.05, **P < 0.01, ***P < 0.001, ****P < 0.0001.

## Supplementary information

Supplementary Figures and Tables

Supplementary Genelist

Supplement for Figure 1 501Mel ChIP

Supplement for Figure 1 COLO829 ChIP

Supplement for Figure 1 Melanocyte ChIP

Supplement for Figure 1 ChIP overlap

Supplement for Figure 1 ChIP overlap annotations

Supplement for Figure 5 TCGA GO
