# Supplementary material for: MITF has a central role in regulating starvation-induced autophagy in melanoma
Source: Sci Rep. 2019 Jan 31;9:1055. doi: 10.1038/s41598-018-37522-6 (PMC6355916; doi:10.1038/s41598-018-37522-6)
Supplement: Supplementary file 1 — Supplementary Figures and Tables [file 41598_2018_37522_MOESM1_ESM.pdf]

MITF has a central role in regulating starvation-induced autophagy in melanoma

Katrin Möller[1,#], Sara Sigurbjornsdottir[1,#], Asgeir O Arnthorsson[1,#], Vivian Pogenberg[2], Ramile Dilshat[1], Valerie Fock[1], Solveig H Brynjolfsdottir[1], Christian Bindesboll[3], Margret Bessadottir[1], Helga M Ogmundsdottir[1], Anne Simonsen[3], Lionel Larue[4], Matthias Wilmanns[2], Vesteinn Thorsson[5], Eirikur Steingrimsson[1*], Margret H Ogmundsdottir[1].

[1] Department of Biochemistry and Molecular Biology, Biomedical Center, Faculty of Medicine, University of Iceland, Sturlugata 8, 101 Reykjavik, Iceland.

[2] European Molecular Biology Laboratories, Notkestrasse 85, 22761 Hamburg, Germany.

[3] Institute of Basic Medical Sciences and Centre for Cancer Cell Reprogramming, Institute of Clinical Medicine, Faculty of Medicine, University of Oslo, 1112 Blindern, 0317 Oslo, Norway.

[4] Institut Curie, PSL Research University, INSERM U1021, Normal and Pathological Development of Melanocytes Orsay, France; Université Paris-Sud, Université Paris-Saclay, CNRS UMR 3347 Orsay, France; Equipe Labellisée Ligue Contre le Cancer Orsay, France.

[5] Institute for Systems Biology, 401 Terry Avenue North, Seattle, WA 98109, USA.

*Corresponding author; eirikurs@hi.is

#These authors contributed equally to the manuscript.

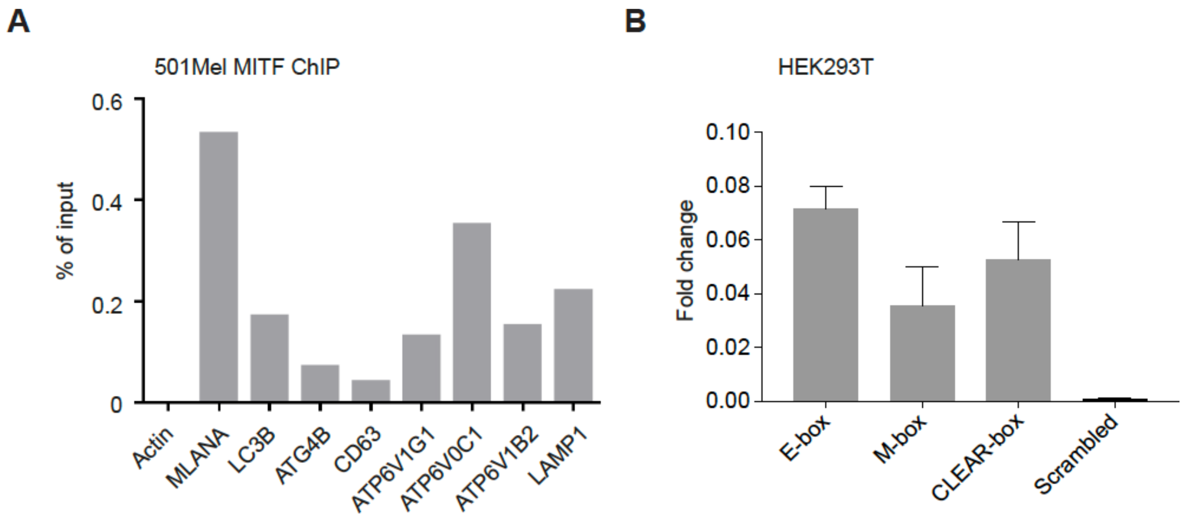

Supplemental Figure 1. MITF binds the promoters of lysosomal and autophagy genes and activates E-, M- and CLEAR-box elements.

A MITF ChIP-qPCR of the indicated genes in 501Mel cells. The % binding of total input is shown. A representative of three replicate experiments is shown.

B Luciferase trans-activation by MITF in HEK293T cells of a modified tyrosinase promoter, containing two E-, M- or CLEAR-box elements, or scrambled elements.

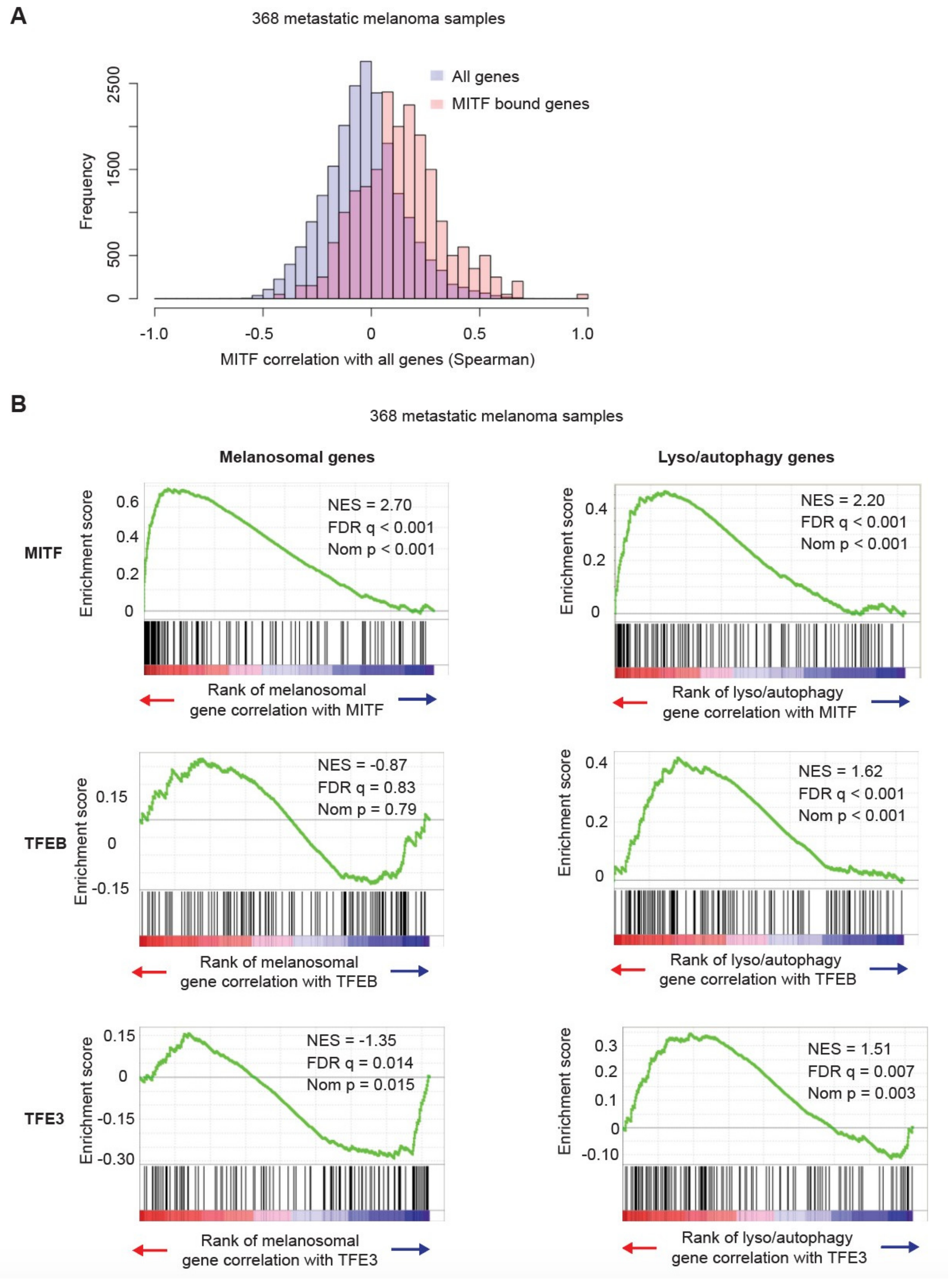

Supplemental Figure 2. ***MITF*** **correlates with lysosomal and autophagosomal gene expression in metastatic melanoma tumors.**

A The light blue histogram shows the distribution of the Spearman correlation coefficient between *MITF* and all other genes, in 368 TCGA metastatic melanoma samples. The beige histogram (pink when it overlaps with blue) shows the histogram of the correlation between *MITF* and 395 genes known to be bound by MITF according to ChipSeq experiments, but amplified 50-fold in the vertical direction, for ease of comparison.

B Genes were ranked according to the correlation of expression with *MITF*, *TFEB* or *TFE3* in 368 metastatic melanoma tumors. GSEA for melanosomal and lysosomal/autophagy genes is shown and Normalized Enrichment Score (NES), False Discovery Rate (FDR) and Nominal p value are indicated.

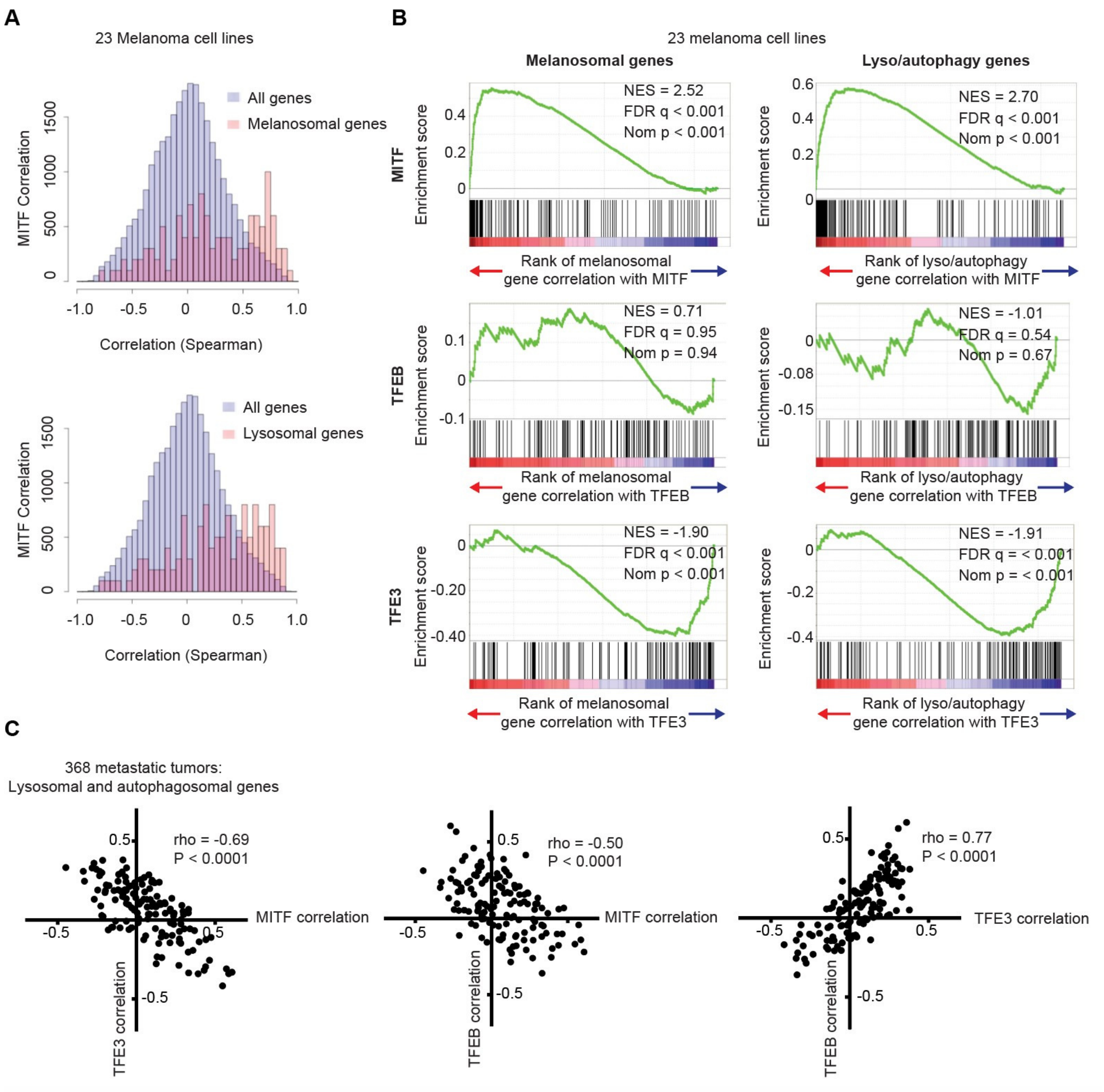

Supplemental Figure 3. *MITF* correlates with lysosomal and autophagosomal gene expression in melanoma cell lines.

A Distribution of the Spearman correlation coefficient for the expression of *MITF* with the expression of all other genes (light blue histogram) in 23 melanoma cell lines, and the analogous correlation for melanosomal genes or lysosomal and autophagosomal genes (beige histogram and pink when overlapping with blue). The melanosomal or lyso/autophagy gene histogram is expanded 100-fold in the vertical direction, to allow easier comparison.

B Genes were ranked according to the correlation of expression with *MITF*, *TFEB* or *TFE3* in 23 melanoma cell lines[33]. GSEA for melanosomal, and lysosomal and autophagosomal genes is shown and Normalized Enrichment Score (NES), False Discovery Rate (FDR) and Nominal p value are indicated.

C Cross comparison of correlation of *MITF*, *TFEB* and *TFE3* expression with lysosomal and autophagosomal gene expression in 368 TCGA metastatic tumor samples.

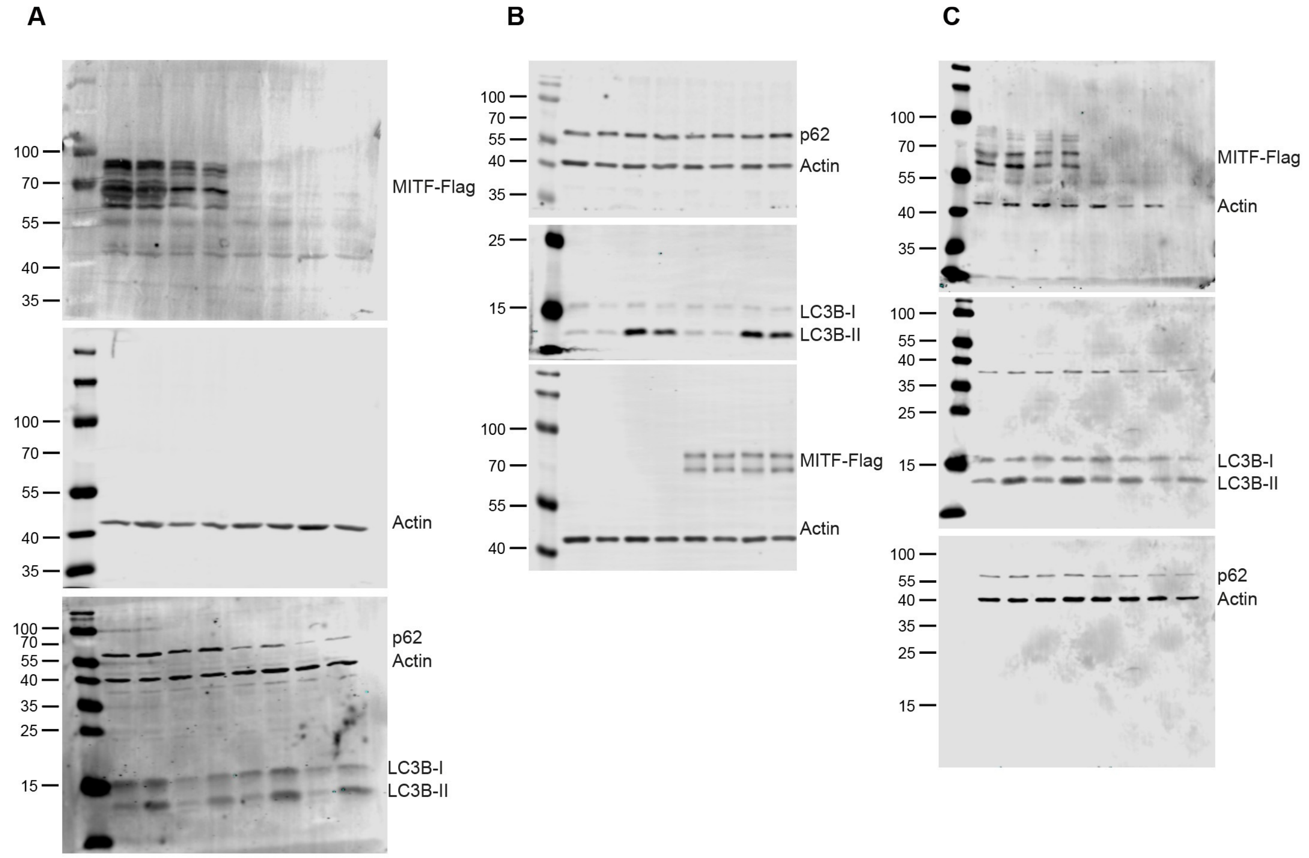

Supplemental Figure 4. Full scans of Western blots presented in main Figures 4D, 5E and Supplemental Figure 5C.

A Western blots presented in Figure 4D. The two upper images show a membrane stained with antibodies against FLAG and Actin, the upper shows a single channel image of Flag, the lower shows a single channel image Actin. The bottom blot shows a membrane stained with antibodies against p62, Actin and LC3B.

B Western blots presented in Figure 5E. The two upper images show a membrane stained with antibodies against p62, Actin and LC3B. The upper image shows a double channel image of p62 and Actin and the lower image shows a single channel image of LC3B, using longer exposure time. The bottom blot shows a membrane stained with antibodies against Flag and Actin.

C Western blots presented in Supplemental Figure 5C. The upper membrane was stained with antibodies against FLAG and Actin. The lower two images represent membranes stained with antibodies against p62, Actin and LC3B, the upper shows a single channel image of LC3B, the lower a single channel image of p62 and Actin.

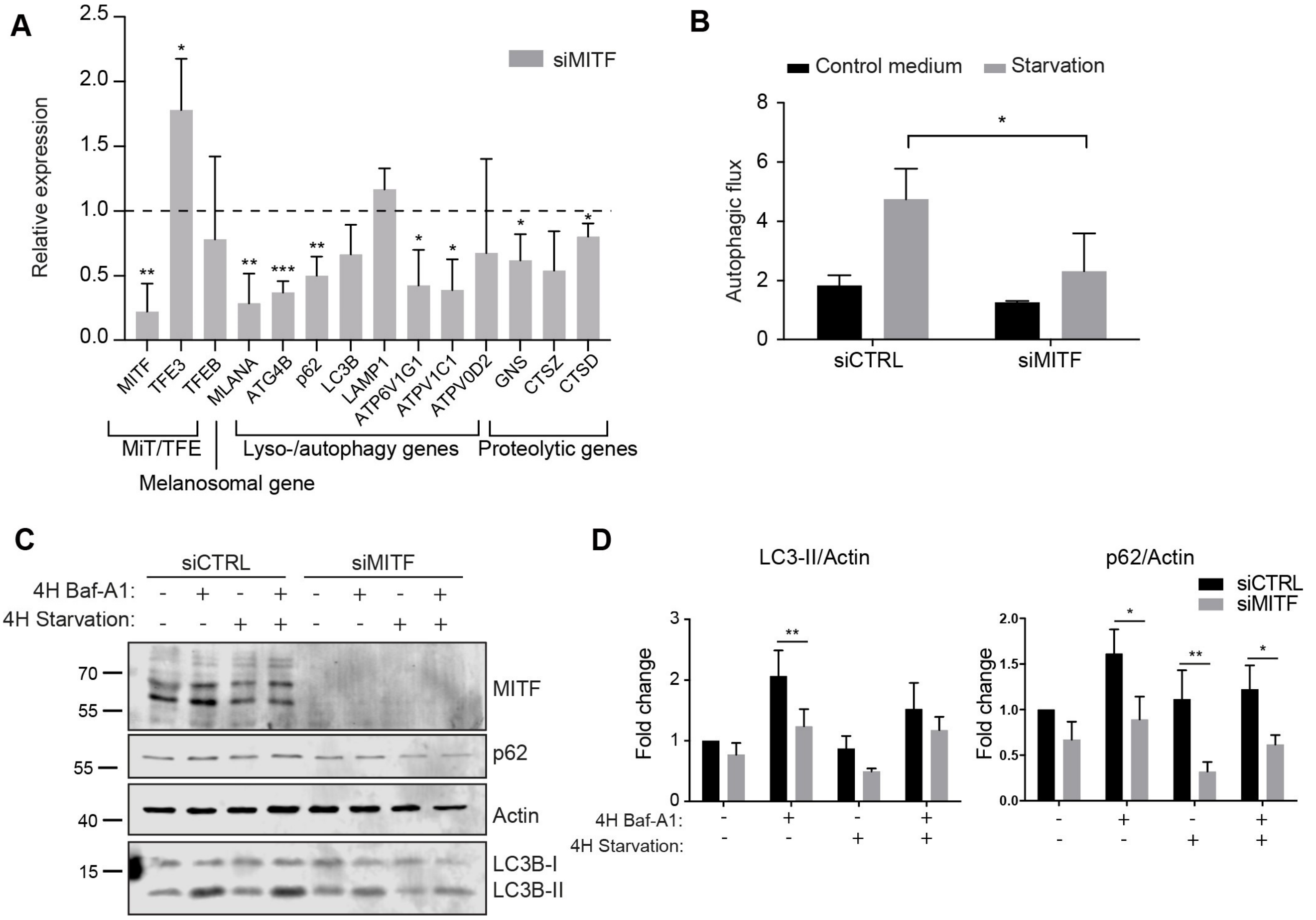

Supplemental Figure 5. MITF regulates starvation-induced autophagy in primary NHEM cells.

A qRT-PCR analysis of selected targets in primary NHEM cells treated with siMITF compared with siCTRL (siCTRL is normalized to 1 for all targets and not shown). An average of 3 independent experiments is shown. Error bars represent SEM, *P<0.05, **P<0.01, ***P<0.001, ****P<0.0001.

B Degradation of long-lived proteins measured by ^{14}C labelled Valine in NHEM cells treated with siMITF compared with control siRNA, treated with or without Baf-A1 while grown in normal culture medium or starved in HBSS for 4 hours. Each bar represents the difference of degradation in untreated and Baf-A1 treated cells, thus representing degradation by autophagy. An average of three independent experiments is shown. Error bars represent SEM, *P<0.05.

C Immunoblot of protein lysate from siMITF and siCTRL treated NHEM cells cultured in normal medium or starved in HBSS for 4 hours with or without 100nM Baf-A1. The blots were stained with C5 anti-MITF, anti-Actin, anti-p62 and anti-LC3B antibodies. The figure is representative of three independent experiments. Full scan of blots are found in Supplemental Figure 4.

D Quantification of the intensity of LC3B-II and p62 bands from immunoblot in C, normalized to Actin band intensity. An average of three independent experiments is shown. Error bars represent SEM, **P<0.01.

Supplemental Table 1. X-ray structure determination.

**Data collection**	
Space group	$I2_12_12_1$
Unit cell dimensions [Å]	a = 44.1, b = 103.9, c = 112.9
Wavelength [Å]	0.966
Resolution range [Å] [a]	40.58-2.40 (2.49-2.40)
Unique reflections	10,159 (1087)
Multiplicity [a]	4.4 (4.2)
Mean $I/\sigma_{(I)}$ [a]	12.1 (1.7)
Completeness [%] [a]	99.5 (100.0)
Half-set correlation ($CC_{1/2}$)	0.99 (0.60)
R_{sym} [b]	5.9 (76.3)
**Refinement**	
Number of protein atoms	1005
R_{cryst}[%] [c,d]	21.5
R_{free} [%] [e]	25.3
RMSD bond lengths [Å]	0.008
RMSD angles [°]	1.33
Mean B-factors [$Å^2$]	0.856
Ramachandran plot:	
Favored regions [%]	98.7
Clash score (Chen et al., 2010)	5

[a] Values in parentheses refer to the highest resolution shell.

[b] $R_{sym}(I) = \Sigma_{hkl}\Sigma_i | I_{hkl,i} - \langle I_{hkl,i}\rangle | / \Sigma_{hkl}\Sigma_i | I_{hkl,i} |$ where $\langle I_{hkl,i}\rangle$ is the mean intensity of symmetry-related measurements of the reflections.

[c] $R_{cryst} = \Sigma_{hkl} | F_{obs} - F_{calc}| / \Sigma_{hkl} | F_{obs} |$

[d] R_{free} is as R_{cryst} but calculated over 9 % of data that were excluded from the refinement process.

[e] RMSD is the root-mean square deviation from ideal geometry.

Supplemental Table 2. Comparison of mean expression of MiT/TFEs in TCGA tumor samples (368 metastatic melanomas) and a panel of human melanoma cell lines (23 cell lines).

	**MITF**	**TFEB**	**TFE3**	**TFEC**	**MITF/TFEB Fold difference**	**MITF/TFE3 Fold difference**
Metastatic	5947	437	1442	144	13.6	4.1
Cell lines	67183	1435	1273	79	46.8	52.8

Units are displayed as RSEM http://www.biomedcentral.com/1471-2105/12/323

Supplemental Table 3. Gene Set Enrichment analysis of TCGA data and melanoma cell lines showing correlation values for MITF, TFEB and TFE3.

	Melanosomal genes			Lyso/autophagy genes		
	**MITF**	**TFEB**	**TFE3**	**MITF**	**TFEB**	**TFE3**
**Metastatic tumors**	2.70 ($p<0.001$)	-0.87 ($p=0.79$)	-1.35 ($p=0.015$)	2.20 ($p<0.001$)	1.62 ($p<0.001$)	1.51 ($p=0.003$)
**Cell lines**	2.52 ($p<0.001$)	0.71 ($p=0.94$)	-1.90 ($p<0.001$)	2.70 ($p<0.001$)	-1.01 ($p=0.67$)	-1.91 ($p<0.001$)

Supplemental Table 4. Gene ontology analysis on top 2,000 positively correlated genes with MITF, TFEB or TFE3 in 368 metastatic melanoma samples.

GO term	p-value
MITF top 2,000 positively correlated genes in metastatic melanoma tumors	
GO:0006099~tricarboxylic acid cycle	1.13×10^{-8}
GO:0042438~melanin biosynthetic process	3.50×10^{-7}
GO:0030318~melanocyte differentiation	4.75×10^{-7}
GO:0015031~protein transport	1.53×10^{-6}
GO:0006886~intracellular protein transport	2.68×10^{-6}
GO:0042787~protein ubiquitination involved in ubiquitin-dependent protein catabolic process	7.53×10^{-6}
GO:0055114~oxidation-reduction process	7.92×10^{-6}
GO:0016050~vesicle organization	2.93×10^{-5}
GO:0016236~macroautophagy	7.97×10^{-5}
TFEB top 2,000 positively correlated genes in metastatic melanoma tumors	
GO:0006955~immune response	1.21×10^{-56}
GO:0006954~inflammatory response	2.29×10^{-54}
GO:0002250~adaptive immune response	3.94×10^{-33}
GO:0045087~innate immune response	1.33×10^{-28}
GO:0050776~regulation of immune response	7.31×10^{-27}
GO:0031295~T cell costimulation	4.01×10^{-23}
GO:0060333~interferon-gamma-mediated signaling pathway	4.41×10^{-22}
GO:0070098~chemokine-mediated signaling pathway	5.52×10^{-19}
GO:0007165~signal transduction	4.32×10^{-18}
TFE3 top 2,000 positively correlated genes in metastatic melanoma tumors	
GO:0006954~inflammatory response	2.06×10^{-51}
GO:0006955~immune response	1.60×10^{-51}
GO:0007155~cell adhesion	3.17×10^{-22}

GO:0006935~chemotaxis	3.49×10^{-19}
GO:0030198~extracellular matrix organization	8.98×10^{-19}
GO:0050776~regulation of immune response	3.67×10^{-17}
GO:0050900~leukocyte migration	7.49×10^{-17}
GO:0001525~angiogenesis	4.29×10^{-16}
GO:0007165~signal transduction	7.22×10^{-15}

Supplemental Table 5. Primers used for qRT-PCR

Target	Primer	Sequence
MITF	FW	5'-ATGGAAACCAAGGTCTGCCC-3'
	REV	5'-GGGAAAAATACACGCTGTGAGC-3'
β-Actin	FW	5'-AGGCACCAGGGCGTGAT-3'
	REV	5'-GCCCACATAGGAATCCTTCTGAC-3'
RPLP0	FW	5'-CACCATTGAAATCCTGAGTGATGT-3'
	REV	5'-TGACCAGCCCAAAGGAGAAG -3'
TFEB	FW	5'-AAGGAGCGGCAGAAGAAAGA-3'
	REV	5'-CCAACTCCTTGATGCGGTCA-3'
TFE3	FW	5'-CAGCTGCTCAGCCTGAACTC-3'
	REV	5'-CTTGAGCGAAGGGGTAAGGG-3'
MLANA	FW	5'-TGGATACAGAGCCTTGATGGATAA-3'
	REV	5'-GAGACACTTTGCTGTCCCGA-3'
LC3B	FW	5'-CCGCACCTTCGAACAAAGAG-3'
	REV	5'-AAGCTGCTTCTCACCCTTGT-3'
ATG4B	FW	5'-TCCTGAACCTGTCCCTAGATTC-3'
	REV	5'-CCCGACCCCAGGATTTTCAA-3'
LAMP1	FW	5'-CACCATCCAGGCGTACCTTT-3'
	REV	5'-TGTTCACAGCGTGTCTCTCC-3'
ATP6V1G1	FW	5'-GGCTAGTCAGTCTCAGGGGA-3'
	REV	5'-CCGGTTCTTTCTTTTGCGGG-3'
CTSZ	FW	5'-GCTATGGCGGATCGGATCAA-3'
	REV	5'-TACCGCAGTCGATGACGTTC-3'
GNS	FW	5'-TTGCCCATTTTGAGAGGTGC-3'
	REV	5'-CAGTGACGTTACGGCCTTCT-3'
CTSD	FW	5'-TCAGGGCGAGTACATGATCC-3'
	REV	5'-GGGGACAGCTTGTAGCCTTT-3'
ATP6V1C1	FW	5'-TTGCATGCGGCAACTTCAAA-3'
	REV	5'-CGTGCCAACCTTTAAGTCAGG-3'
ATP6V0D2	FW	5'-TTCTTGAGTTTGAGGCCGACA-3'
	REV	5'-TGGATAGAGGGTCTCTCGGT-3'

Supplemental Table 6. ChIP-qRT-PCR primers

Target	Primer	Sequence
MLANA	FW	5’-TGGGTTCTTCCAATGTGTCA-3‘
	REV	5’-TTTATGCATGGTCACGTGGT-3‘
β-Actin	FW	5’-AGTGTGGTCCTGCGACTTCTAAG-3‘
	REV	5’-CCTGGGCTTGAGAGGTAGAGTGT-3‘
ATG4B	FW	5’-CTTCCCTTTGCTGCCTCCT-3‘
	REV	5’-GAAGGCTGTAACACTGTGGC-3‘
CD63	FW	5’-TGCCCCATATCACAAGTGGT-3‘
	REV	5’-ACCGAGAATGGCCATCCTG-3‘
LC3B	FW	5’-ACAGCCACCAGGAGAGTTCC-3‘
	REV	5’-CTTTTGTCCCGAGCCTTCAT-3‘
LAMP1	FW	5’-TCACTTTCTCCCGCCACTAC-3‘
	REV	5’-GAGGTCCTGAAACGGGAAGT-3‘
ATP6V1G1	FW	5’-TCCTCTCTTGACGTTGAGCA-3‘
	REV	5’-CTACCCTGTCGCTGGTTCAC-3‘
ATP6V0C	FW	5’-AAGTGGTACGGCTCGCAG-3‘
	REV	5’-GCTCTAAATACCAGCACCGC-3‘
ATP6V1B2	FW	5’-GGCGATCTGAAGCCTGACTA-3‘
	REV	5’-TCCACCGAATCAAATGTTTTC-3‘
